# *ALDH2* rs671 and *MTHFR* rs1801133 polymorphisms are risk factors for arteriosclerosis in multiple arteries

**DOI:** 10.1186/s12872-023-03354-0

**Published:** 2023-06-24

**Authors:** Nan Cai, Cunren Li, Xianfang Gu, Wenfeng Zeng, Jingfeng Liu, Guopeng Zeng, Jiawei Zhong, Junxing Zhu, Haifeng Hong

**Affiliations:** 1 Center for Cardiovascular Diseases, Meizhou People’s Hospital, Meizhou Academy of Medical Sciences, No. 63 Huangtang Road, Meijiang District, Meizhou, China; 2 Guangdong Provincial Engineering and Technology Research Center for Molecular Diagnostics of Cardiovascular Diseases, Meizhou, China

**Keywords:** *ALDH2*, *MTHFR*, Arteriosclerosis, Multiple arteries

## Abstract

**Background:**

Arteriosclerosis in multiple arteries has long been associated with heightened cardiovascular risk. Acetaldehyde dehydrogenase 2 (ALDH2) and methylenetetrahydrofolate reductase (MTHFR) play an important role in the pathogenesis of arteriosclerosis by participating in the oxidation and reduction reactions in vascular endothelial cells. The purpose was to investigate the relationship of *ALDH2* and *MTHFR* gene polymorphisms with arteriosclerosis in multiple arteries.

**Methods:**

410 patients with arteriosclerosis in single artery and 472 patients with arteriosclerosis in multiple arteries were included. The relationship between *ALDH2* rs671 and *MTHFR* rs1801133 polymorphisms and arteriosclerosis in single artery and arteriosclerosis in multiple arteries was analyzed.

**Results:**

The proportion of *ALDH2* rs671 A allele (35.6% vs. 30.9%, *P* = 0.038) and *MTHFR* rs1801133 T allele (32.6% vs. 27.1%, *P* = 0.012) in patients with arteriosclerosis in multiple arteries was significantly higher than that in arteriosclerosis in single artery, respectively. The proportion of history of alcohol consumption in patients with *ALDH2* rs671 G/G genotype was higher than those in *ALDH2* rs671 G/A genotype and A/A genotype (*P* < 0.001). The results of logistic regression analysis indicated that *ALDH2* rs671 A/A genotype (A/A vs. G/G: OR 1.996, 95% CI: 1.258–3.166, *P* = 0.003) and *MTHFR* rs1801133 T/T genotype (T/T vs. C/C: OR 1.943, 95% CI: 1.179–3.203, *P* = 0.009) may be independent risk factors for arteriosclerosis in multiple arteries (adjusted for age, sex, smoking, drinking, hypertension, and diabetes).

**Conclusions:**

*ALDH2* rs671 A/A and *MTHFR* rs1801133 T/T genotypes may be independent risk factors for arteriosclerosis in multiple arteries.

## Introduction

Arteriosclerosis refers to the accumulation of fatty and/or fibrous material in the intima of arteries, and it remains a major killer, and has now spread globally [1, 2]. Arteriosclerosis refers to the aging and structural changes of the arterial system. In terms of pathology, arteriosclerosis is mainly manifested by elastin fatigue fracture, collagen deposition and crosslinking, and cholesterol deposition in the artery wall as the main pathological basis, leading to fibrous tissue hyperplasia and calcinosis of the affected artery, and gradually metamorphosis and calcification, resulting in thickening and hardening of the artery wall and narrowing of the vascular cavity [3–5]. Arteriosclerosis is an intermediate lesion connecting cardiovascular risk factors and cardiovascular events, and is the risk factor for cardiovascular and cerebrovascular diseases [6, 7]. Prevention of arteriosclerosis can delay the occurrence of cardiovascular and cerebrovascular events [8]. As a systemic disease, arteriosclerosis in multiple arteries is defined as arteriosclerosis within 2 or more arterial beds, it has long been associated with heightened cardiovascular risk [9, 10]. Arteriosclerosis often involves multiple arterial beds simultaneously and causes different clinical symptoms, and different sites of the arteries may have different degrees of arterial disease in the same patient, suggesting that risk factors for arteriosclerosis may have different degrees of effect on different sites of the arteries [11, 12].

Studies have found that the occurrence of arteriosclerosis is related to some risk factors, such as older age, male gender, hypertension, diabetes, hyperlipidemia, smoking, and drinking [13, 14], and may also be related to some genetic factors [15]. The occurrence of arteriosclerosis is associated with oxidative stress [16]. Oxidative stress at the cellular level may produce toxic aldehydes, and excess aldehydes are extremely toxic to cells [17]. Acetaldehyde dehydrogenase (ALDH) family participate in the oxidation and metabolism of aldehydes in the body [18], and ALDH2 is the most widely studied in ALDH family [19]. ALDH2 is a mitochondrial protein containing 517 amino acids, encoded by the *ALDH2* gene [20], and the most common polymorphism in *ALDH2* gene is Glu504Lys polymorphism (single nucleotide polymorphism (SNP) rs671, G1510A) [21]. At the SNP rs671, guanine (G) is replaced by adenine (A), resulting in the encoded amino acid from glutamic acid (Glu) to lysine (Lys). The mutation of this site changes the structure of ALDH2, and the binding of coenzyme NAD (P) + with the mutant ALDH2 is impaired, and the activity of ALDH2 is reduced due to the weakened dehydrogenation [22]. *ALDH2* Glu504Lys mutation leads to the decrease of ALDH2 activity and the accumulation of aldehydes, which is associated with the occurrence of various diseases [23].

Homocysteine (Hcy) promotes programmed death of coronary endothelial cells and accelerates the occurrence of arteriosclerosis [24]. 5-10-Methylenetetrahydrofolate reductase (MTHFR) catalyses the irreversible reduction of 5-10-MTHF to 5-methylTHF, a circulatory form of folate used in the remethylation of Hcy to methionine [25]. The decrease of MTHFR activity affects the metabolic process of Hcy and leads to the increase of Hcy level that damages vascular endothelium [26]. In addition, nitric oxide (NO) can enhance vasodilatation and reducing platelet aggression and adhesion in vascular endothelium, which plays an important role in homeostasis around vascular endothelium [27, 28]. Levels of Hcy and MTHFR, play a determining role in circulating levels of NO [27]. *MTHFR* C677T (rs1801133) is the most common polymorphism of *MTHFR* gene, and the mutant allele was associated with high level of Hcy [29]. *MTHFR* C677T gene is mutated from cytosine (C) to thymine (T) at base 677, thereby changing codon 222 from alanine (Ala) to valine (Val). This region is the base binding site of flavin adenine dinucleotide (FAD), thus altering enzyme activity [30].

To sum up, ALDH2 and MTHFR play an important role in the pathogenesis of arteriosclerosis by participating in the oxidation and reduction reactions of vascular endothelial cells. Study has found that the mutated *ALDH2* carriers were more susceptible to multi-coronary artery lesions in Chinese patients with CAD [31]. The *MTHFR* rs1801133 polymorphism was risk factor for carotid artery arteriosclerosis [32]. But the result of the relationship of *ALDH2* and *MTHFR* gene polymorphisms and arteriosclerosis in multiple arteries is still unclear. The different regions, populations, lifestyles and gene polymorphisms will affect the development of arteriosclerosis [33–35]. In the current study, we evaluated the association between *ALDH2* rs671, *MTHFR* rs1801133 polymorphisms and arteriosclerosis in single artery and arteriosclerosis in multiple arteries. This study will provide valuable information for guiding screening of patients at risk for multiple sites of arteriosclerosis.

## Materials and methods

### Study Population

A total of 882 unrelated individuals were included in this retrospective study, including 410 patients with arteriosclerosis in single artery and 472 patients with arteriosclerosis in multiple arteries, collected from Meizhou People’s Hospital, China between January 2016 and July 2019. This study was approved by the Ethics Committee of Meizhou People’s Hospital.

Arteriosclerosis is determined by tests such as angiography, magnetic resonance imaging (MRI), or computed tomography evaluated by two senior radiologists in a double-blind evaluation. In this study, arteriosclerosis was observed in coronary artery, carotid artery, cerebral artery, and limb artery. The patients’ medical records were collected from the Hospital Information System (HIS) of Meizhou People’s Hospital, including age, sex, smoking history, alcohol consumption history, hypertension, diabetes, and arteriosclerosis.

### Genotyping of *ALDH2* rs671 and *MTHFR* rs1801133

Genomic DNA was extracted from whole blood using a QIAamp DNA Blood Mini Kit (Qiagen GmbH, North Rhine-Westphalia, Germany) according to the manufacturer’s protocol. *ALDH2* rs671 polymorphism and *MTHFR* rs1801133 polymorphism was genotyped by *ALDH2* and *MTHFR* genotyping kit (BaiO Technology Co, Ltd, Shanghai, China), respectively. PCR was performed according to the following protocol: denaturation at 94℃ for 5 min; amplification of 35 cycles (94℃ for 25 s, 56℃ for 25 s, and 72℃ for 25 s); final elongation at 72℃ for 5 min. The PCR amplification product was hybridized with the probe fixed on the chip, and the specific hybridization signal was chromogenic by enzyme chromogenic reaction.

### Statistical analysis

Data analysis was performed using SPSS statistical software version 21.0 (IBM Inc., USA). Student’s t-test or the Mann-Whitney U test was used for continuous data analysis. Genotype composition ratios and allele frequencies of groups were analyzed by the χ^2^ test. The χ^2^ test was used to test the significance of the Hardy-Weinberg equilibrium (HWE) of the *ALDH2* rs671 and *MTHFR* rs1801133 polymorphisms in the entire data of the patients with arteriosclerosis in single artery and patients with arteriosclerosis in multiple arteries. To measure the relative risk of *ALDH2* rs671 and *MTHFR* rs1801133 genotypes in arteriosclerosis, logistic regression analysis was performed after adjusting for sex, age, smoking history, alcohol consumption history, hypertension, and diabetes. The statistical significance level of all analysis results was defined as a *P* < 0.05.

## Results

### Patient demographics

A total of 882 patients were studied in this study, of which 308 (34.9%) were younger than 65 years old and 574 (65.1%) were ≥ 65 years old. There were 605 male patients (68.6%) and 277 female patients (31.4%). There were 410 patients with arteriosclerosis in single artery (46.5%) and 472 patients with arteriosclerosis in multiple arteries (53.5%) in this study. The differences in age distribution, sex distribution, proportions of patients with history of smoking and alcohol consumption, and proportions of patients with hypertension and diabetes between the patients with arteriosclerosis in single artery and patients with arteriosclerosis in multiple arteries were not statistically significant (all *P* > 0.05) (Table 1).

**Table 1:** Clinical characteristics of patients with arteriosclerosis in single artery and patients with arteriosclerosis in multiple arteries

	Total (n = 882)	Patients with arteriosclerosis in single artery (n = 410)	Patients with arteriosclerosis in multiple arteries (n = 472)	*P* values
Age, years	69.05 ± 10.82	69.28 ± 11.33	68.86 ± 10.37	
< 65, n(%)	308 (34.9)	138 (33.7)	170 (36.0)	0.479
≥ 65, n(%)	574 (65.1)	272 (66.3)	302 (64.0)	
Sex				
Male, n(%)	605 (68.6)	281 (68.5)	324 (68.6)	1.000
Female, n(%)	277 (31.4)	129 (31.5)	148 (31.4)	
History of smoking, n(%)	201 (22.8)	86 (21.0)	115 (24.4)	0.260
History of alcohol consumption, n(%)	38 (4.3)	14 (3.4)	24 (5.1)	0.247
Hypertension, n(%)	576 (65.3)	263 (64.1)	313 (66.3)	0.524
Diabetes, n(%)	285 (32.3)	133 (32.4)	152 (32.2)	0.943

### Frequencies of *ALDH2* rs671 and *MTHFR* rs1801133 genotypes in patients with arteriosclerosis in single artery and arteriosclerosis in multiple arteries

The χ^2^ test was used to test the significance of the Hardy-Weinberg equilibrium of the *ALDH2* rs671 and *MTHFR* rs1801133 polymorphisms in the patients with arteriosclerosis in single artery and arteriosclerosis in multiple arteries. The distributions of *ALDH2* rs671 genotypes in patients with arteriosclerosis in single artery (χ^2^ = 0.492, *P* = 0.483) and patients with arteriosclerosis in multiple arteries (χ^2^ = 1.551, *P* = 0.213) were consistent with Hardy-Weinberg equilibrium, respectively. The distributions of *MTHFR* rs1801133 genotypes in patients with arteriosclerosis in single artery (χ^2^ = 0.069, *P* = 0.793) and patients with arteriosclerosis in multiple arteries (χ^2^ = 0.025, *P* = 0.875) were consistent with Hardy-Weinberg equilibrium, respectively. The frequencies of *ALDH2* rs671 and *MTHFR* rs1801133 genotypes and alleles were compared between the arteriosclerosis in single artery and arteriosclerosis in multiple arteries groups. The proportion of *ALDH2* rs671 A allele in patients with arteriosclerosis in multiple arteries was significantly higher than that in patients with arteriosclerosis in single artery (35.6% vs. 30.9%, *P* = 0.038). The proportion of *MTHFR* rs1801133 T allele in patients with arteriosclerosis in multiple arteries was significantly higher than that in patients with arteriosclerosis in single artery (32.6% vs. 27.1%, *P* = 0.012) (Table 2).

**Table 2:** Frequencies of *ALDH2* rs671, *MTHFR* rs1801133 genotypes and alleles in patients with arteriosclerosis in single artery and patients with arteriosclerosis in multiple arteries

Genotype/allele	Total (n = 882)	Patients with arteriosclerosis in single artery (n = 410)	Patients with arteriosclerosis in multiple arteries (n = 472)	*P* values
*ALDH2* rs671				
G/G	395(44.8%)	193(47.1%)	202(42.8%)	0.047
G/A	385(43.7%)	181(44.1%)	204(43.2%)	
A/A	102(11.6%)	36(8.8%)	66(14.0%)	
G	1175(66.6%)	567(69.1%)	608(64.4%)	0.038
A	589(33.4%)	253(30.9%)	336(35.6%)	
HWE (χ^2^, *P*)	χ^2^ = 0.308, *P* = 0.579	χ^2^ = 0.492, *P* = 0.483	χ^2^ = 1.551, *P* = 0.213	
*MTHFR* rs1801133				
C/C	432(49.0%)	217(52.9%)	215(45.6%)	0.039
C/T	370(42.0%)	164(40.0%)	206(43.6%)	
T/T	80(9.1%)	29(7.1%)	51(10.8%)	
C	1234(70.0%)	598(72.9%)	636(67.4%)	0.012
T	530(30.0%)	222(27.1%)	308(32.6%)	
HWE (χ^2^, *P*)	χ^2^ = 0.004, *P* = 0.951	χ^2^ = 0.069, *P* = 0.793	χ^2^ = 0.025, *P* = 0.875	

HWE, Hardy Weinberg Equilibrium

The number of the subjects with *ALDH2* rs671 G/G, G/A, and A/A genotype was 395(44.8%), 385(43.7%), and 102(11.6%), respectively, with the number of the subjects with *MTHFR* rs1801133 C/C, C/T, and T/T genotype was 432(49.0%), 370(42.0%), and 80(9.1%), respectively. While clinical characteristics were compared among subjects stratifed by *ALDH2* rs671 genotypes, the proportion of history of alcohol consumption in patients with *ALDH2* rs671 G/G genotype was higher than those in patients with *ALDH2* rs671 G/A genotype and A/A genotype (*P* < 0.001). The differences in other clinical characteristics among the different *ALDH2* rs671 genotypes were not statistically significant. In addition, the differences in all clinical characteristics among the different *MTHFR* rs1801133 genotypes were not statistically significant (Table 3).

**Table 3:** Clinical characteristics of subjects stratified by *ALDH2* rs671 and *MTHFR* rs1801133 genotypes

Clinical characteristics	*ALDH2* rs671				*MTHFR* rs1801133			
	G/G (n = 395)	G/A (n = 385)	A/A (n = 102)	*P* values	C/C (n = 432)	C/T (n = 370)	T/T (n = 80)	*P* values
Age, years								
< 65, n(%)	142(35.9%)	134(34.8%)	32(31.4%)	0.685	152(35.2%)	132(35.7%)	24(30.0%)	0.633
≥ 65, n(%)	253(64.1%)	251(65.2%)	70(68.6%)		280(64.8%)	238(64.3%)	56(70.0%)	
Sex								
Male, n(%)	265(67.1%)	265(68.8%)	75(73.5%)	0.452	294(68.1%)	252(68.1%)	59(73.8%)	0.588
Female, n(%)	130(32.9%)	120(31.2%)	27(26.5%)		138(31.9%)	118(31.9%)	21(26.3%)	
History of smoking, n(%)	88(22.3%)	91(23.6%)	22(21.6%)	0.862	99(22.9%)	86(23.2%)	16(20.0%)	0.817
History of alcohol consumption, n(%)	32(8.1%)	6(1.6%)	0(0)	< 0.001	22(5.1%)	13(3.5%)	3(3.8%)	0.531
Hypertension, n(%)	267(67.6%)	245(63.6%)	64(62.7%)	0.427	280(64.8%)	244(65.9%)	52(65.0%)	0.944
Diabetes, n(%)	127(32.2%)	122(31.7%)	36(35.3%)	0.782	138(31.9%)	122(33.0%)	25(31.3%)	0.936

### Association of the risk factors with arteriosclerosis in multiple arteries

To gain insight into the independent risk factors on arteriosclerosis in multiple arteries, logistic regression analysis was performed. The possible association of the *ALDH2* genotypes with potential risk factors for arteriosclerosis in multiple arteries based on three genetic modes of inheritance: co-dominant model, dominant model, and recessive model. The results of univariate logistic regression showed that *ALDH2* rs671 A/A genotype (A/A vs. G/G: odds ratio (OR) 1.752, 95% confidence interval (CI): 1.115–2.751, *P* = 0.015) may increase risk of arteriosclerosis in multiple arteries, and *MTHFR* rs1801133 T/T genotype (T/T vs. C/C: OR 1.775, 95% CI: 1.084–2.907, *P* = 0.023) may increase risk of arteriosclerosis in multiple arteries.

The results of multivariate logistic regression (adjusted for age, sex, smoking history, drinking history, hypertension, and diabetes) indicated that *ALDH2* A/A genotype in the co-dominant model (*ALDH2* A/A vs. *ALDH2* G/G) (adjusted OR 1.996, 95% CI 1.258–3.166, *P* = 0.003) and *ALDH2* A/A genotype in the recessive model (*ALDH2* A/A vs. *ALDH2* G/G + G/A) (adjusted OR 1.802, 95% CI 1.168–2.781, *P* = 0.008) were significant risk factors for the presence of arteriosclerosis in multiple arteries. And the *MTHFR* T/T genotype in the co-dominant model (*MTHFR* T/T vs. *MTHFR* C/C) (adjusted OR 1.943, 95% CI 1.179–3.203, *P* = 0.009), *MTHFR* C/T and T/T genotypes in the dominant model (*MTHFR* C/T plus *MTHFR* T/T vs. *MTHFR* C/C) (adjusted OR 1.387, 95% CI 1.061–1.813, *P* = 0.017), and *MTHFR* T/T genotype in the recessive model (*MTHFR* T/T vs. *MTHFR* C/C plus *MTHFR* C/T) (adjusted OR 1.685, 95% CI 1.042–2.723, *P* = 0.033) were significant risk factors for the presence of arteriosclerosis in multiple arteries (Table 4).

**Table 4:** Association of the risk factors with arteriosclerosis in multiple arteries

Variables			Univariate OR (95% CI)	*P* values	Multivariate OR (95% CI)	*P* values
Age (≥ 65/<65)			0.901(0.683–1.190)	0.464	0.930(0.696–1.242)	0.621
Sex (Male/ Female)			0.995(0.748–1.323)	0.973	1.127(0.826–1.539)	0.450
History of smoking (Yes/No)			1.214(0.884–1.667)	0.232	1.226(0.858–1.752)	0.263
History of alcohol consumption (Yes/No)			1.515(0.773–2.970)	0.226	1.727(0.854–3.495)	0.128
Hypertension (Yes/No)			1.100(0.833–1.453)	0.500	1.152(0.866–1.533)	0.331
Diabetes (Yes/No)			0.989(0.746–1.313)	0.941	0.986(0.738–1.318)	0.925
*ALDH2* rs671						
	Co-dominant	G/G	1.000(reference)		1.000(reference)	
		G/A	1.077(0.813–1.426)	0.606	1.159(0.869–1.546)	0.315
		A/A	1.752(1.115–2.751)	0.015	1.996(1.258–3.166)	0.003
	Dominant	G/G	1.000(reference)		1.000(reference)	
		G/A + A/A	1.189(0.911–1.551)	0.203	1.278(0.972–1.681)	0.079
	Recessive	G/G + G/A	1.000(reference)		1.000(reference)	
		A/A	1.689(1.099–2.595)	0.017	1.802(1.168–2.781)	0.008
*MTHFR* rs1801133						
	Co-dominant	C/C	1.000(reference)		1.000(reference)	
		C/T	1.268(0.959–1.675)	0.095	1.317(0.993–1.747)	0.056
		T/T	1.775(1.084–2.907)	0.023	1.943(1.179–3.203)	0.009
	Dominant	C/C	1.000(reference)		1.000(reference)	
		C/T + T/T	1.344(1.031–1.752)	0.029	1.387(1.061–1.813)	0.017
	Recessive	C/C + C/T	1.000(reference)		1.000(reference)	
		T/T	1.592(0.988–2.563)	0.056	1.685(1.042–2.723)	0.033

## Discussion

The result of the relationship of *ALDH2* and *MTHFR* gene polymorphisms and arteriosclerosis in multiple arteries is still unclear. In the current study, we evaluated the association between *ALDH2* rs671, *MTHFR* rs1801133 polymorphisms and arteriosclerosis in single artery and arteriosclerosis in multiple arteries. The results of this study show that *ALDH2* rs671 A/A and *MTHFR* rs1801133 T/T genotypes may be independent risk factors for arteriosclerosis in multiple arteries.

*ALDH2* rs671 A/A genotype may be an independent risk factor for arteriosclerosis in multiple arteries. Study has shown that individuals with *ALDH2* rs671 G/A or A/A genotype have a higher coronary artery disease (CAD) risk than individuals with G/G genotype [36]. Another study found that the mutated *ALDH2* carriers were more susceptible to multi-vessel lesions [31]. In the perspective of mechanism, ALDH2 plays an important role in the development and progression of arteriosclerosis by inhibiting oxidative low-density lipoprotein (ox-LDL)-induced foam cell formation via suppressing CD36 (cluster of differentiation 36) expression [37]. It has found that the appropriate dose of ethanol reduced arterial plaque formation in ApoE-/- mice, while ALDH2 deficiency blocked the protection of ethanol against arterial plaque formation [38]. Another animal study has shown that ALDH2 inhibits transcription of a lysosomal proton pump protein ATP6V0E2 (ATPase H(+) Transporting V0 Subunit E2) to degrade ox-LDL, while ALDH2 encoded by *ALDH2* rs671 polymorphism weakens this effect [39].

*MTHFR* rs1801133 T/T genotype may be an independent risk factor for arteriosclerosis in multiple arteries. Studies have found that *MTHFR* rs1801133 polymorphism was significantly associated with peripheral arterial disease (PAD) [40, 41]. The *MTHFR* rs1801133 polymorphism was risk factor for carotid artery arteriosclerosis [32]. The *MTHFR* rs1801133 polymorphism was associated with increased risk of CAD in different populations [42–46]. In mechanism, vascular 5-methyl-tetrahydrofolate (5-MTHF) is a key regulator of human vascular endothelial nitric oxide synthase coupling and nitric oxide bioavailability, and *MTHFR* rs1801133 polymorphism plays an important role in the regulation of vascular redox status by affecting the expression of vascular 5-MTHF [47].

To our knowledge, this study is the first report of the relationship of *ALDH2* rs671 and *MTHFR* rs1801133 genotypes and arteriosclerosis in multiple arteries. Our study found that *ALDH2* rs671 A/A and *MTHFR* rs1801133 T/T genotypes may be independent risk factors for arteriosclerosis in multiple arteries. It is of great significance for the screening and prevention of high risk individuals with arteriosclerosis in multiple arteries. Early screening for mutations in the *ALDH2* and *MTHFR* genes should be done in people who have already found single atherosclerosis.

There are some limitations in present study. First, no information was collected about the degree or grade of arteriosclerosis of the subjects, which limited the analysis of the relationship between *ALDH2* rs671 and *MTHFR* rs1801133 and the degree or grade of arteriosclerosis. Second, this study only analyzed the relationship between the common polymorphisms of *ALDH2* and *MTHFR* genes and the risk of arteriosclerosis in multiple arteries. Third, deficiencies in folic acid can increase homocysteine levels, induce endothelial dysfunction, and accelerate pathological process of arteriosclerosis [48] and MTHFR is an enzyme involved in the metabolism of folic acid [49]. This study did not examine the role of *MTHFR* polymorphism in arteriosclerosis formation in people with low folic acid levels. So, future studies that include larger sample sizes, the degree of arteriosclerosis, the analysis of the full-length variation of *ALDH2* and *MTHFR* genes, and analysis of folic acid levels are needed.

## Conclusion

In summary, the relationship between *ALDH2* rs671 and *MTHFR* rs1801133 polymorphisms and arteriosclerosis in multiple arteries was identified in a cohort study. After adjusting age, sex, history of smoking and drinking, hypertension, and diabetes, *ALDH2* rs671 A/A and *MTHFR* rs1801133 T/T genotypes may be independent risk factors for arteriosclerosis in multiple arteries. Of course, further research is needed to verify our results and investigate the mechanism of the reported association.

## Data Availability

The datasets used and analyzed during the current study available from the corresponding author on request.
